# Short-Term *In Vitro* ROS Detection and Oxidative Stress Regulators in Epiretinal Membranes and Vitreous from Idiopathic Vitreoretinal Diseases

**DOI:** 10.1155/2022/7497816

**Published:** 2022-12-16

**Authors:** Bijorn Omar Balzamino, Lucia Dinice, Andrea Cacciamani, Agnese Re, Fabio Scarinci, Luca Bruno, Pamela Cosimi, Alessandra Micera

**Affiliations:** ^1^Research Laboratories in Ophthalmology, IRCCS-Fondazione Bietti, Rome, Italy; ^2^Retinal Unit, IRCCS-Fondazione Bietti, Rome, Italy; ^3^Università Cattolica del Sacro Cuore, Rome, Italy

## Abstract

**Background:**

A plethora of inflammatory, angiogenic, and tissue remodeling factors has been reported in idiopathic epiretinal membranes (ERMs). Herein we focused on the expression of a few mediators (oxidative, inflammatory, and angiogenic/vascular factors) by means of short-term vitreal cell cultures and biomolecular analysis.

**Methods:**

Thirty-nine (39) ERMs and vitreal samples were collected at the time of vitreoretinal surgery and biomolecular analyses were performed in clear vitreous, vitreal cell pellets, and ERMs. ROS products and iNOS were investigated in adherent vitreal cells and/or ERMs, and iNOS, VEGF, Ang-2, IFN*γ*, IL18, and IL22 were quantified in vitreous (ELISA/Ella, IF/WB); transcripts specific for *iNOS*, *p65NFkB*, *KEAP1*, *NRF2*, and *NOX1/NOX4* were detected in ERMs (PCR). Biomolecular changes were analyzed and correlated with disease severity.

**Results:**

The higher ROS production was observed in vitreal cells at stage 4, and iNOS was found in ERMs and increased in the vitreous as early as at stage 3. Both *iNOS* and *NOX4* were upregulated at all stages, while *p65NFkB* was increased at stage 3. *iNOS* and *NOX1* were positively and inversely related with *p65NFkB*. While *NOX4* transcripts were always upregulated, *NRF2* was upregulated at stage 3 and inverted at stage 4. No significant changes occurred in the release of angiogenic (VEGF, Ang-2) and proinflammatory (IL18, IL22 and IFN*γ*) mediators between all stages investigated.

**Conclusions:**

ROS production was strictly associated with *iNOS* and *NOX4* overexpression and increased depending on ERM stadiation. The higher *iNOS* expression occurred as early as stage 3, with respect to *p65NFkB* and *NRF2*. These last mediators might have potential prognostic values in ERMs as representative of an underneath retinal damage.

## 1. Introduction

The epiretinal membranes (ERMs) are weak membranes placed over the retina, between the retinal nerve fiber layer and vitreous [1–4]. These transparent, hypocellular, and avascular extracellular matrix-based ERMs can produce a tension on the underneath retinal network, with wrinkling and/or swelling [5, 6]. The long-lasting ERM-contractile activity might trigger structural and functional macular changes (edema), foveal dystopia with variable clinical symptoms, and visual acuity impairments [2]. Genetic background and ageing, epigenetic, metabolic, life-styling, and other environmental influences partake in the development of ERMs and modulate the overall retraction [7].

ERMs come from the proliferation and migration of inflammatory cells within the retinal pigment epithelium (RPE) and glial (Müller cells, microglia, and fibrous astrocytes) layers, providing a scaffold for hyalocytes and macrophages [4, 8]. A most recent hypothesis displays that an insufficient dehiscence of the liquefied vitreous body with the vitreoretinal interface induces a break in the posterior vitreous cortex (vitreoschisis), leaving the posterior vitreous cortex layer attached to the macula. This can be partially dependent on idiopathic or iatrogenic derivation [8].

In a recent study, our group analyzed the whole-flattened ERMs at histological, biochemical, and molecular levels, identifying some inflammatory mediators and tissue matrix factors [9]. A plethora of inflammatory, angiogenic, and tissue-remodeling factors was quantified in ERMs and related vitreal fluids, suggesting that the long-lasting inflammation and matrix retraction might be responsible for altered homeostasis and switch to parainflammation and release of reactive oxygen species (ROS) and reactive nitrogen species (RNS) [10, 11]. ROS production was recently detected in ERMs and vitreal fluids from diabetic patients, prospecting a possible contribution in proliferative vitreous retinopathy (PVR) [12]. Retinal ganglion cells (RGCs), Müller cells, astrocytes, and microglia are elective producers of ROS metabolites [13, 14]. In addition, ROS/RNS ratio has been prospected in the modulation of tissue contraction/retraction, representing an additional field in ERM severity. ROS products are strictly dependent on inducible Nitric Oxide (NO) Synthase (*iNOS*) and the activity of Nicotinamide Adenine Dinucleotide Phosphate (*NADPH*) enzymes, NADPH Oxidase 1 (*NOX1*) and NADPH Oxidase 4 (*NOX4*). Recent attention has been devoted to some neuroprotective mechanisms driven by Kelch-like ECH-associated protein 1 (*KEAP1*) and Nuclear Factor- (erythroid-derived 2-) like 2 (*NRF2*), two key nuclear transcription factors involved in systemic and local antioxidant defense system [15].

Therefore, the aim of this study was to (i) investigate the potential intracytoplasmatic ROS production by vitreal cells using a short-term cell culture approach; (ii) characterize the biomolecular expression of *iNOS* and *KEAP1*/*NRF2* factors and *NOX1*/*NOX4* in ERM tissues and (iii) the expression of few selected mediators in the vitreal fluids, all collected at the time of therapeutic surgery and representative of different stages of disease severity. This investigation would be of interest for future drug-design, targeting some mediators that might take part in the process of distress of the retina, induced by contractile ERM activity. The biochemical analysis of the vitreous, as representative of an underneath retinal damage, might have potential prognostic values as previously prospected [16–18].

## 2. Materials and Methods

The study was approved by the Intramural Ethical Committee (IFO-Bietti, Rome, Italy) and performed in accordance with the ethical standards stated in the Declaration of Helsinki.

### 2.1. Study Population and ERM Grading

A total of thirty-nine patients (39; 31F/8M; 71.00 ± 6.35 years old) were recruited before therapeutic surgery and grouped according to disease severity (*n* = 12/stage 2; *n* = 14/stage 3; *n* = 13/stage 4). Demographic, clinical information, and samples (vitreous/ERMs) were collected in patients providing a written-informed consent, as approved by the Ethical Committee. The inclusion criteria comprised of adult patients diagnosed for ERM and selected for therapeutical vitrectomy [19]. The exclusion criteria included patients with ERM at stage 1 or with macular holes, patients receiving anti-VEGF intravitreal treatments or topical antiglaucoma therapy, subjects undergoing eye surgery in the past or retinal laser therapy in the last 3 months prior to surgery, intraocular pressure (IOP) higher than 22 mmHg, and comorbidities such as systemic neurodegenerative diseases (Alzheimer's or Parkinson's diseases) or local/systemic autoimmune diseases (merely Sjogren's Syndrome and diabetes) as well as any vascular, degenerative, or inflammatory diseases.

Anamnesis, funduscopic evaluation, and spectral domain-optical coherence tomography (Spectralis SD-OCT ver.1.5.12.0; Heidelberg Engineering, Heidelberg, Germany) and disease staging and ERM grading were carried out at the visit for recruitment. Patients provided written adherence to the protocol by signing the informed consent.

### 2.2. Vitreous and ERMs: Biosample Management

Sampling was performed at the time of routine 25-gauge pars-plana vitrectomy [20]. Vitreous was first collected followed by peeled-off ERMs, and both samples were quickly delivered to the laboratory. Four subgroups were produced according to ERM severity (Govetto's classification) as follows: stage 2, ERMs associated with widening of nuclear layer and loss of foveal depression; stage 3, ERMs associated with continuous ectopic inner foveal layers crossing the entire foveal area; stage 4, thick ERMs, association with continuous ectopic inner foveal layers and severe disruption of retinal layers [19]. Patients defined as stage 1 (*n* = 0), including mild and thin ERMs with presence of foveal depression, were not included in the study, as not eligible for surgery [19].

Pure vitreous (250-500 *μ*L) was quickly centrifuged at 2000 rpm for 7 min (1-14 microfuge; Sigma, St. Louis, Missouri, USA) to separate “floating” cells from the clear fluid. Clarified vitreous were supplemented with 1 *μ*L protease inhibitors/sample (Pierce, Thermo-fisher Scientific, Waltham, Massachusetts, USA) and quickly sonicated (VibraCell; Sonics, Newtown, CT) to sprinkle residual cells or free nucleic acids (RNA/DNA) and further centrifuged (13000 rpm/7 min) to remove residual debris. Spectrophotometric analysis was performed on 3 *μ*L extracted samples (Nanodrop; Celbio, EuroClone S.p.A, Milano, Italy) before producing aliquots for biochemical analysis.

Peeled-off ERMs were removed and placed on pretreated glass-slides (BDH, Milan, Italy) postfixed with BioFix (BioOptica, Inc., Milano, Italy) and stored until epifluorescent microscopy and molecular analysis. ERM specimens were processed in lysis buffer to extract simultaneously total RNA and proteins (mirVana-PARIS™ RNA and Native Protein Purification Kit; Thermo Fisher Scientific).

### 2.3. Vitreal Cells: Intracellular ROS Visualization and Quantification

Vitreous samples were quickly delivered to the laboratory and after a 1 : 2 dilution in Hank's Balanced Sodium Salt (HBSS), vitreous samples were placed on special 8-well slides (Nunc™ Lab-Tek II™ 8 wells; Thermo Scientific™) to let the adherence of vitreal cells to the slides (37°C for 30 min with 5% CO_2_). After gentle vitreous aspiration, adherent cells were exposed to a cell permeant reagent 2′,7′–dichlorofluorescin diacetate (H2DCFDA) working solution, according to the manufacturers' instructions (ab113851; DCFDA/H2DCFDA-Cellular ROS Assay Kit; Abcam, Cambridge, UK). Washed cells were thereafter counterstained with DAPI prepared in Phosphate Buffered Saline (PBS, 10 mM PB and 137 mM NaCl; pH 7.5; Invitrogen-Molecular Probes, Eugene, Oregon). Fluorescent cells (Ch1/green) having blue nuclei (Ch3/blue) were observed at the inverted Eclipse TE2000U microscope, and images were acquired by C1 software (Nikon, Tokyo, Japan). Channel series were carried out to reduce autofluorescence. Digital images (pixel size: 1024 × 1024 dpi) were converted into 8-bit TIFF images and subjected to densitometric analysis (ImageJ v1.43; http://rsb.info.nih.gov/ij/). Single integrated optical density (IntDen) was registered for fluorescent ROS expression at different stages (*n* = 5, optic fields/slide; ×40/dry 0.75 DIC M/N2), mean values ± SD were used for statistical analysis.

### 2.4. Microscopical Analysis: Double Immunostaining and Digital Acquisitions

Prefixed whole mounted ERMs were briefly equilibrated in PBS and blocked/permeabilized with 0.1% BSA/0.3% Triton X-100 in PBS before adding anti-human iNOS antibodies developed in rabbits (1 : 100; Abcam). Secondary Cy2/green conjugated anti-rabbit specie-specific F(ab)2 antibodies (1 : 500-1 : 700 in 0.05% Tween20-PBS) were added for 45 min/benchtop (Jackson Research Laboratories, West Grove, PA). Nuclear visualization was performed while mounting ERMs (blue/DAPI; Invitrogen-Molecular Probes, Eugene, Oregon) in antifading PBS solution. Examinations were carried out under epifluorescent direct microscope (Ni-Eclipse; Nikon) equipped with UV lamp (Nikon), digital camera (Axiocam 208 color; Carl Zeiss, Jena, Germany), and the free available ZEN 3.1 acquisition software (blue edition). Single specific acquisitions were carried out at ×20 objectives and merge was performed according to a standard procedure (8-TIFF format).

### 2.5. Biochemical Analysis: ELISA and Ella Microfluidics

*ELISA*. Aliquots (50 *μ*L/sample) were 1 : 2 diluted in sample dilution buffer provided by commercially available VEGF-A ELISA kits including precoated 96-well plates and ready-to-use solutions (EH2VEGF; Thermo-fisher Scientific). Absorbance (Optical Density-OD) values were recorded after plate reading (*λ*450-*λ*570 nm) and concentrations were produced according to standard curves (assay range: 31.25-2000 pg/mL; sensitivity ≤ 5 pg/mL)

*Ella Microfluidics*. Aliquots (25 *μ*L samples) were 1 : 2 diluted and loaded onto customized cartridges for analysis in automated multiplex platform designed for Ang2, IFN*γ*, IL18, and IL22 detection (Protein Simple, CA, USA). Cartridges included built-in lot specific standard curves, and samples were provided as triplicates. Single mean values (pg/mL) were automatically calculated and provided as “xls format” for statistical analysis.

### 2.6. Molecular Analysis: RNA Extraction, cDNA Synthesis, and Amplifications

Total RNA was extracted from ERMs (*n* = 18) according to the mirVana-PARIS and dissolved in 11 *μ*L RNAse-free water (DEPC-treated and autoclaved MilliQ water, Millipore, Waltham, Massachusetts, USA). A routine spectrophotometric analysis (1.5 *μ*L total RNA per sample) was carried out for RNA quantification/assessment of quality (Nanodrop, Thermo-fisher Scientific). Retro-transcription (100 ng total RNA) was carried out by using the ExcelRT Reverse Transcription polymerase (SMOBIO Technology, Inc., Hsinchu City, Taiwan) in the presence of dNTPs and random primers (Promega, Milan, Italy). Protocol of cDNA synthesis was performed in a LifePro Thermal Cycler (EuroClone, Milan, Italy). cDNAs (3 *μ*L/target and 1 *μ*L/referring gene) were amplified using the Hydra SYBR Green hot start PCR Master Mix (Biocell, Rome, Italy) in Eco Real-Time PCR System (Illumina Inc., San Diego, CA, USA), in parallel with negative controls. Cq values (Illumina) from normalized samples showing one melting curve were run in REST program. Changes in gene expression at stages 3 and 4 were provided as log2 expression ratio with respect to stage 2 (referring group), considering the 18S house-keeping gene. Primer pairs were synthesized by Eurofin MWG Genomics (https://eurofinsgenomics.eu/) and summarized in Table 1.

Accession numbers (GeneBank) were reported as by NCBI search and amplicon length ranged between 100-250 bps. Amplification procedure was as follows: initial hot start activation (95°C/5 min) followed by 39 cycles of denaturation (94°C/10s)/annealing (58-60°C/15 s)/extension (75°C/10s) and melting curve generation (58°C-95°C with one fluorescence reading every 0.5°C).

### 2.7. Statistics

To satisfy the assumption of data coming from a normally distributed population, row values were analyzed by the Kolmogorov-Smirnov and the Shapiro-Wilk tests (Prism9.4; GraphPad Software Inc., San Diego, CA). ANOVA analysis was used to compare protein expression between subgroups, while the REST-ANOVA coupled analysis was carried out for identifying significant changes in real time PCR experiments. Correlations were assessed by using the free-download available R studio for windows. A *p* < 0.05 was considered statistically significant.

## 3. Results

**Figure 1:**
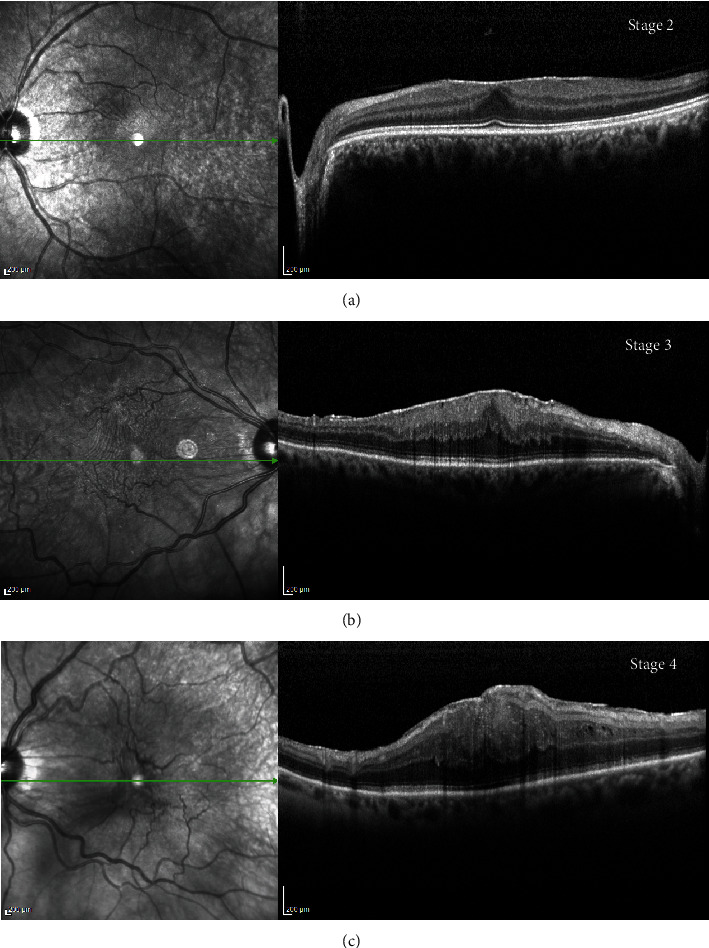
Representative infrared (left) and related spectral domain-optical coherence tomography (OCT, right) images showing the epiretinal membrane (ERM) layered over retinal tissues. Note the ERM traction causing foveal distortion and alterations of the inner retinal structures in all stages investigated, according to the Govetto classification systems. (a) Stage 2, ERM with flattened foveal contour; (b) stage 3, ERM with the presence of ectopic inner foveal layer, and (c) stage 4, ERM with disorganization of inner and outer retinal layers and macular disruption. Green line indicates the examined region; scale bar = 200 *μ*m.

Complete ophthalmic examination was performed before surgery and disease severity was defined and used for categorizing biosamples. Representative images from OCT analysis of the three disease stages investigated are shown in Figure 1. The presence of ERMs layered over retinal tissues and ERM traction causing foveal distortion and alterations of the inner retinal structures in all stages investigated are visible.

Both vitreous and peeled-off ERMs were collected at the time of pars-plana vitrectomy and were subjected to analysis to verify biomolecular changes related to the major oxidative stress and angiogenic and inflammatory protein profiles and transcript modulators.

### 3.1. Differences in Intracellular ROS in Vitreal Cells Depending on Disease Severity

**Figure 2:**
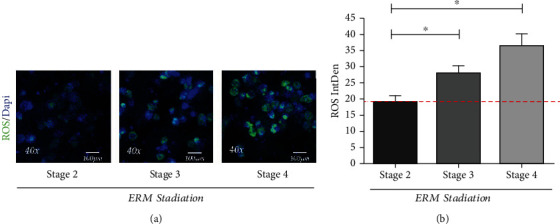
Intracellular ROS immunoreactivity in short-term cultured vitreal cells depending on ERM severity. Adherent cells were evaluated for the ability to produce ROS products by using the Cellular ROS Assay Kit. Not-pooled samples were used for H2DCFDA assay. Fluorescent intensities were monitored and acquired. (a) Representative confocal images of intracellular ROS in vitreous adhering cells at different stages of disease progression. (b) Bar plots showing the increased ROS expression depending on ERM severity. IntDen, Integrated Density. ANOVA analysis followed by the Tukey-Kramer post hoc highlighted the significant effects indicated in the graphs, ^∗^*p* ≤ 0.05.

To understand the ROS production inside the vitreal chamber, short-term cultures of vitreal cells were developed from vitreal fluids. An increasing immunofluorescent signal specific for ROS was observed intracellularly upon exposure to a substrate (see MM section). As shown in Figure 2(a), the intracellular ROS immunoreactivity (green) was high in vitreal cells from stage 3 (27.56 ± 5.28 IntDen; *p* < 0.05) and particularly from stage 4 (36.29 ± 7.60 IntDen; *p* < 0.05), as compared to stage 2 (18.69 ± 4.37 IntDen). Intracellular ROS production was quantified according to the ImageJ software (IntDen measurements) and the results of quantification are shown in Figure 2(b).

### 3.2. iNOS Protein Increases in Peeled-off ERMs and Vitreal Fluids

**Figure 3:**
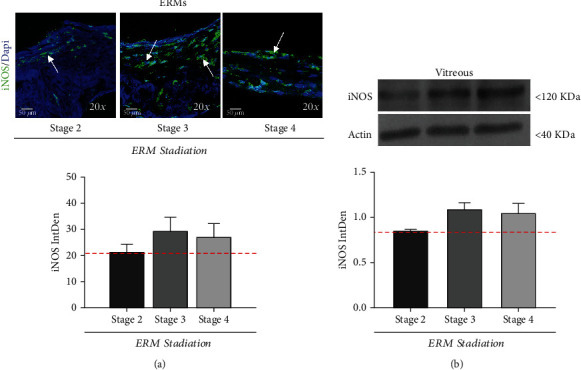
iNOS protein changes as function of disease severity. Epifluorescent and Western Blot analyses followed by densitometric analysis (below: ImageJ, IntDen). (a) ERMs. Representative epifluorescent images and IntDen histogram displaying the immunoreactivity of iNOS protein depending on ERM severity. Merged (green/blue) panels of iNOS (green) over a DAPI counterstaining (blue nuclei). Scale bar = 50 *μ*m (b) vitreous. Vitreal iNOS immunoblotting and related iNOS band quantitation (120 kDa expected size). Immunoblots were destained and reprobed against *β*-actin (40 kDa expected band). Values are mean ± SD from 3 independent experiments carried out in triplicate.

Whole-flattened ERMs were used for verifying the presence of cellular iNOS immunoreactivity. The presence of several cell subsets inside the fibrocellular-matrix compartment was confirmed by nuclear staining (DAPI/blue) of ERMs. Cellularity was found significantly reduced at stage 4. As shown by arrows in Figure 3(a), the number of immunoreactive iNOS-positive cells (green/cy2; ×20) were increased at stage 3 and stage 4 with respect to stage 2 (*p* ≥ 0.05). The respective vitreous samples were analyzed for iNOS protein expression by Western Blotting. A specific iNOS increase (120 kDa expected size) was observed in ERMs at stage 3 and stage 4 with respect to stage 2 (*p* ≥ 0.05), as supported by the IntDen quantification of specific bands (Figure 3(b)). Stripped immunoblots were reprobed with *β*-actin (40 kDa expected band) for IntDen normalization purposes.

### 3.3. Transcripts for *iNOS*, *p65NFkB*, and Some Oxidative Stress Regulators Are Differentially Expressed in ERMs

**Figure 4:**
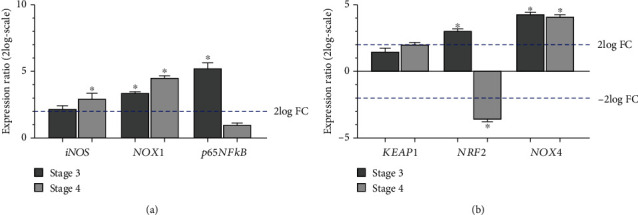
Inflammatory and oxidative stress regulators' transcripts in ERMs. Relative expression ratio (fold-changes; mean ± SD; log2-scale) in ERMs at stage 3 and stage 4, with respect to stage 2 (Rest-ANOVA Tukey-Kramer's coupled analysis). (a) Histogram showing a significant upregulation for *iNOS* (stage 4), *NOX1* (stage 3 and stage 4), and *p65NFkB* (stage 3) transcripts, with respect to stage 2 (^∗^*p* < 0.05). (b) Significant changes in the transcripts' expression were observed in *NRF2* (upregulation at stage 3 and deregulation and stage 4) and *NOX4* (upregulation at stage 3 and stage 4), as calculated with respect to stage 2 (^∗^*p* < 0.05).

The expressions of *iNOS*, *NOX1*, and *p65NFkB* and *KEAP1*, *NRF2*, and *NOX4* transcripts were investigated in total RNAs from ERMs at different stages of disease. Relative PCR analysis showed the upregulation of *iNOS* transcripts at stage 4 and *NOX1* transcripts at stage 3 and particularly at stage 4, with respect to stage 2. Of interest, the expression of *p65NFkB* transcripts was increased selectively at stage 3 while not significant changes were observed at stage 4, with respect to stage 2 (Figure 4(a)).

The analysis of epigenetic targets showed no significant changes for *KEAP1* at all stages (Figure 4(b); *p* > 0.05) while *NRF2* transcripts were increased at stage 3 and decreased at stage 4 (Figure 4(b); *p* < 0.05). *NOX4* transcripts were upregulated in ERMs at both disease stages (Figure 4(b); *p* < 0.05).

**Figure 5:**
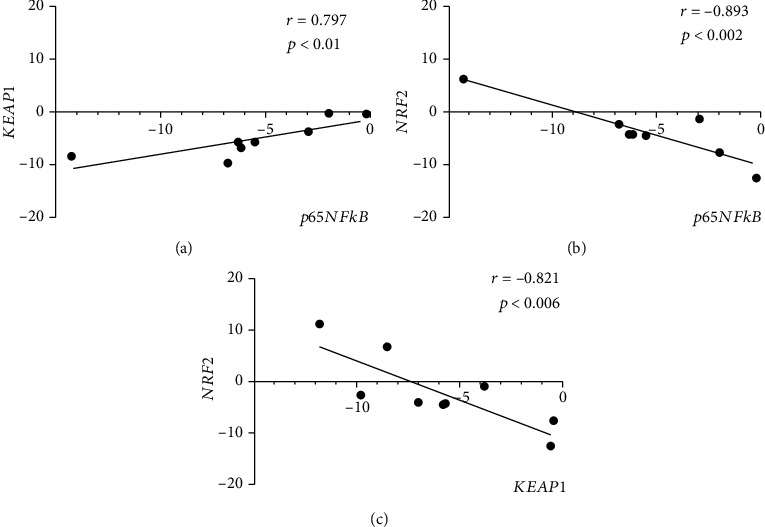
Correlation between *p65NFkB*, *KEAP1*, and *NRF2* upon disease severity. Plots showing the correlation between the inflammatory transcription factor (*p65NFĸB*) and the epigenetic genes *KEAP1* (a) and *NRF2* (b) and between *NRF2* and *KEAP1* (c). rho and *p* values are shown in the panels (Pearson's rho test analysis).

Pearson's rho test analysis showed that *p65NFkB* transcript expression (inflammatory path) correlated positively with *KEAP1* (rho = 0.797; *p* < 0.01; Figure 5(a)) and negatively with *NRF2* (rho = −0.893; *p* < 0.002; Figure 5(b)). Both *KEAP1* and *NRF2* showed a strong inverse relation (rho = −0.821; *p* < 0.006; Figure 5(c)).

### 3.4. VEGFA, Ang-2, IFN*γ*, IL18, and IL22 Are Increased in Vitreous upon ERM Staging

**Figure 6:**
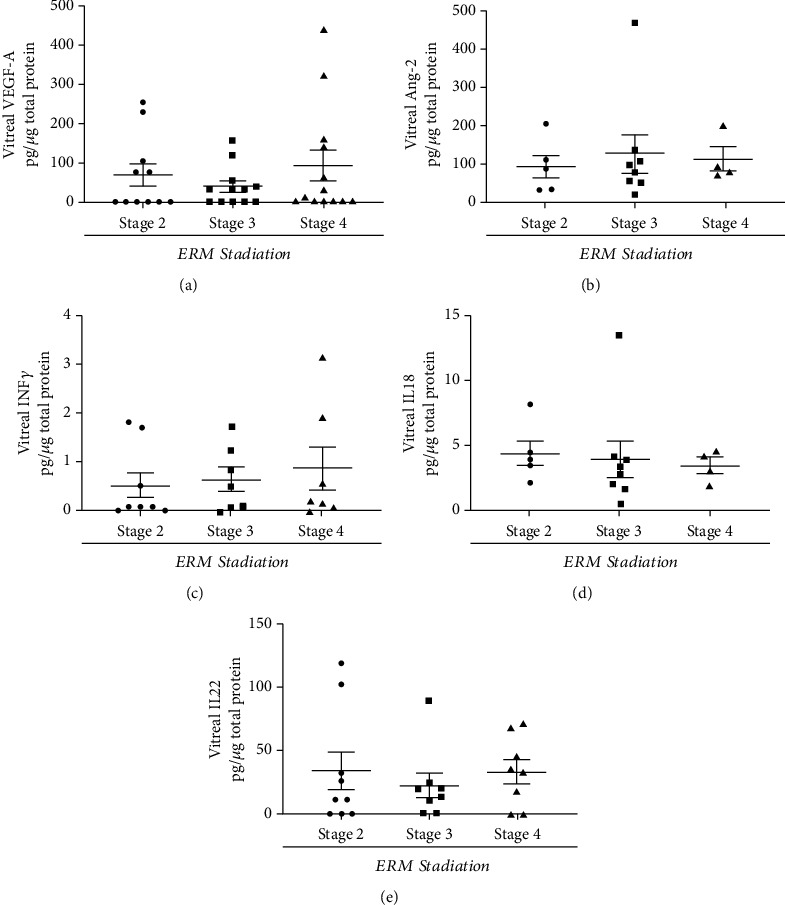
VEGF, Ang-2, IFN*γ*, IL18, and IL22 expression in vitreal samples. Untouched (cleared) vitreal samples were diluted and analyzed for VEGFA (a), Ang-2 (b), IFN*γ* (c), IL18 (d), and IL22 (e) protein expression and compared for ERM staging. As shown by scatter plots, no significant changes were detected in vitreal fluids depending on ERM severity/activation (pg/mg total protein).

Few selected mediators of vitreoretinal disorders were quantified in vitreal samples by conventional (ELISA) and new generation (ELLA) assays. No significant changes were observed for all mediators that were detected in the majority of vitreal samples, as displayed by scatter plots (Figure 6(a)–6(e)).

## 4. Discussion

This study highlights that (i) the expression of iNOS protein and the release of ROS products are increased, respectively, in clear vitreous, vitreal cells, and ERMs; (ii) *NOX1*, *KEAP1*, *NRF2*, and *NOX4* transcripts are differentially expressed in ERMs; finally, (iii) VEGF-A, Ang-2, IFN*γ*, IL-18, and IL-22 proteins were accumulated in vitreal samples, although no significant changes were monitored at different stages of disease.

ERM peel-off still represents the elective therapeutic surgery in case of idiopathic, iatrogenic, or secondary to metabolic/neurodegenerative vitreoretinal diseases showing ERM over retinal tissue [14]. Although the pathogenesis behind ERM formation is still not entirely clarified, the possibility of targeting glial and fibroblast-like cells has been suggested to counteract the local inflammation and the plethora of mediators released in the vitreal chamber [14]. ERMs originate by the activation, proliferation and migration of specific cells localized at the inner surface of the retina and located between the RGC layer and vitreous [21]. Glial cells, mast cells, hyalocytes, and macrophages, in concert with insulted retinal neurons and RPE cells, can modulate the local microenvironment by releasing inflammatory, toxic, profibrogenic, and oxidative products, allowing and/or sustaining the contractile ERM abilities [4, 21, 22]. Up to date, ROS products and some representative mediators of oxidative, inflammatory, and angiogenic pathways have been reported as major players in the inflammatory process occurring at the vitreoretinal interphase [14, 22, 23]. Soluble mediators can trigger the ROS/RNS and NO/iNOS generation by activated immune and structural cells, causing oxidative stress and tissue injury, fulfilling an exacerbation of vitreoretinal retraction (vitreomacular involvement) [11, 22]. By using an indirect *in vitro* method, we observed that short-term cultured vitreal cells, particularly at stage 4, were more capable to produce intracellular ROS products with respect to stage 2 and stage 3, suggesting a strong dependence on disease severity. The high ROS immunoreactivity at later stages might have found an explanation in the cell subtypes populating the ERM formations [14]. Since ROS are produced by activated cells and accumulate intracellularly, ROS are quickly released in the microenvironment, these *in vitro* findings confirm the release of ROS products inside the vitreal chamber by activated cells (activated Müller cells, reactive astrocytes, and resident ameboid macrophages) and justify the development of reactive gliosis and/or scavenger activities as well as potential neuroprotective routes against initial ROS and toxic mediators' release, as previously reported in [14, 23]. In other studies, the production of ROS was directly linked to the expression of *iNOS* and *NOX1*, involved in cellular migration [24–26], the presence of iNOS was investigated in ERMs and related pathological vitreous. The increased iNOS immunoreactivity at early stages of disease and the persisting *iNOS* transcript upregulation at stage 4 highlight a dynamic aspect of iNOS modulation of ERM microenvironment [27]. The local increase of iNOS is usually a consequence of NO overproduction, DNA damaging, reduced cell viability/number, and impaired tissue function, and it is tidily regulated by (i) ROS/RNS, in a dose-dependent fashion, (ii) an interplay of soluble mediators (inflammatory cytokines and transcription factors), and (iii) some matrix enzymes in charge for metabolizing/neutralizing ROS (catalase, glutathione peroxidase, and superoxide dismutase (SODs) [26, 28]. Although we did not verify the specific source of ROS and iNOS inside ERMs, activated glia, myofibroblast-like cells, and endothelial cells might be feasible candidates [4, 29]. In fact, we and other groups reported the presence of *α*SMA-expressing fibroblasts inside ERMs in addition to activated Müller cells, fibrous astrocytes, macrophages, and hyalocytes [9, 21, 29]. Activated glial (GFAP-expressing Müller cells and Iba1-expressing microglia) and endothelial cells showed the ability to respond to matrix/inflammatory stimulators by releasing iNOS in a severe and time-dependent fashion [30]. Moreover, the phase of remodeling—also known as the final phase of tissue healing—is characterized by tissue maturation, retaining functional activity, and reduction of scarring throughout refining of ECM deposition [31]. Apoptotic activity (mainly activated myofibroblasts) and refining of ECM might be reduced by the lower concentration of inflammatory mediators [32]. These aspects have been partially investigated in a previous study of ERM characterization displaying a reduction in cell number and contractile activity at later stages of disease [9].

As second finding, the relationship between *iNOS*, *NOX1*, and *p65NFkB*, as well as *KEAP1*, *NRF2*, and *NOX4* transcripts, strongly support the possibility to have potential direct indicators of disease severity and indirect indicators of retinal senescence, other than cytokines [4, 33]. ROS products are generated in many enzymatic processes (redox reactions) and their prolonged accumulation can exacerbate an established inflammatory process and participate actively in fibrotic processes by driving macrophage polarization and immune senescence, triggering alveolar epithelial cell apoptosis and senescence, promoting myofibroblast differentiation and senescence [24, 25, 34]. As major ROS suppliers, *NOX1* and *p65NFkB* were first investigated, showing a consistent transcript upregulation at later stages. Related to inflammation, *p65NFκB* transcripts were high at stage 3, implying a grade of inflammation, with respect to stage 4 characterized by low cellularity.

To better understand, some tissue-linked transcription factors and ROS modulator enzymes (*iNOS*, *KEAP1*, *NRF2*, and *NOX4*) were analyzed [35, 36]. The high expression of *NOX4* transcripts, an oxidative stress regulator, and the low expression of *NRF2* transcripts at stage 4 would suggest the presence of some antioxidant defense mechanisms working to tune out the dynamic response to oxidative stress, although the reduced cellularity should be also considered [37, 38]. As previously reported, any kind of oxidative stress conditions can display an overexpression of *NRF2* with nuclear translocation, protein recognition elements/dimerization, and nuclear binding to achieve specific gene promoters encoding for antioxidant enzymes [39]. A low cytoplasmatic *NRF2* expression can occur under physiological conditions, in line with *KEAP1*, which in turn is in charge for the physiological *NRF2* proteasome degradation and associated antioxidant defense [35, 40]. To support, an impaired *NRF2* activation in ERMs and association with the retinal cell death was recently reported by other groups [39, 41, 42]. As *NRF2*/*NFκB*-pathways regulate the redox homeostasis, oxidative stress, and inflammatory response, an imbalance between *NRF2* and *NFκB* pathways clearly prompt neurodegeneration, autoimmunity, and tumors, sustaining the neurodegenerating state, as observed in diabetic retinopathy and proliferative vitreoretinopathy associated with ERM development and formation, acting at both transcriptional/post-transcriptional levels inside a specific ERM environment, and reinforcing the neuroinflammatory response, as observed in diabetic retinopathy [43, 44].

A link between *NRF2* and *NOX4* has been prospected in experimental models, highlighting the possibility of a unique redox rheostat response to oxidative stress [45]. On the other side, *NOX4* generates superoxide anions and hydrogen peroxide participating actively to the process [46, 47]. In cardiovascular diseases, an increase of *NRF2* and *NOX4* transcripts has been associated with protective activities against cell death and tissue damage [48]. From *in vitro* studies, the *NOX4*-driven ROS production is regulated by recruitment/activation of *NRF2*, which in turn triggers transcription of an array of antioxidant genes, providing a tidy counteraction of DNA damage, oxidative stress, and caspase 8-mediated apoptosis, with various growth-related responses (angiogenesis/tissue remodeling), and it can reverse fibrosis as observed in the presence of a *NOX4*-*NRF2* redox imbalance, promoting cell senescence and sustaining fibrosis [48–51]. Long-lasting myofibroblast-like cells can produce an altered cell redox homeostasis, resulting from elevated expression of ROS generating enzyme *NOX4* and an impaired capacity to induce the *NRF2* antioxidant response [52, 53].

Finally, a crucial aspect of these vitreoretinal diseases is the local inflammation and angiogenesis and the use of protein signature that has been recently prospected for personalized medicine [18, 54, 55]. These biological fluids are a reservoir of inflammatory mediators, representing potential candidate biomarkers of retinal status [18, 54]. In previous studies, IL1*β* and IFN*γ* were associated with *iNOS* gene activation while *p65NFκB* and *STAT1* were reported for *iNOS* gene transcription [26]. The influence of VEGF-A, IL6, and IL8 (angiogenic factors) and MIP1*α* (leucocyte recruiter molecule) in vitreoretinal disorders has been previously reported for proliferative and/or diabetic retinopathies [17, 56]. Although, VEGF and Ang-2 synergistically influence the local angiogenesis in retinal diseases, we did not observe significant changes between vitreal fluids depending on ERM severity [54–57]. No significant changes were also found for IFN*γ*, IL18, and IL22 (proinflammatory cytokines) depending on disease severity. Since VEGF, Ang-2, IFN*γ*, IL18, and IL22 have been described in vitreoretinal diseases and ERM outgrowth, the presence of outliers at all stages would suggest that a wide study population can biochemically reflect the gliosis, vascular leakage, and neovascularization and inflammation of the underneath retina [58–63].

Although ERM cell population deserves more investigation, our finding about ROS, iNOS, VEGF, Ang-2, *NRF2*, and *NOX4* expression in ERMs and vitreal fluids might represent a key aspect in the developing of individualized therapies [64]. Noteworthy, ERM can slowly progress and deepen the effects on the whole macula, and the risk of developing ERMs rises with ageing and genetic/epigenetic predisposition (ocular and systemic assets) [65]. In this context, it would be of interest to highlight that the entity of inflammation at early stages (at stage 3) might be the explanation of the increased *p65NFkB* mRNA expression, as confirmed by the reduced inflammation and the absence of *p65NFkB* mRNA expression at stage 4. Since oxidative stress is strongly associated with inflammation, *NRF2* transcript parallels the expression of *p65NFkB*[66].

## 5. Conclusions

To date, noninvasive OCT imaging represents the main way to detect the presence and estimate the severity of ERM damage triggered at the underneath central macular zone [67]. Therefore, ROS activation and iNOS expression should be viewed as additional indicators of retinal state and as providing information for local inflammation, metabolic changes, and immune background; together with *NRF2* and *NOX4*, be a sensor for profibrogenic and/or neuroprotective tasks. Genetic and pharmacological targeting of *NOX4* has been previously prospected for testing in animal models of fibrosis to verify the ability to attenuate the senescent-induced fibrosis, antiapoptotic myofibroblast phenotype and to reverse persistent fibrosis [67].

## Figures and Tables

**Table 1:** Primer description.

Genes	Primer sequence		GeneBank
Reference gene			
*18S*	F: GGAGAGGGAGCCTGAGAAAC	R: AGGGCCTCGAAAGAGTCCT	M10098
Target genes			
*iNOS*	F: CCCCTTCAATGGCTGGTACA	R: GTTTCCAGGCCCATTCTCCT	U31511.1
*NOX1*	F: CCAGGATTGAAGTGGATGGT	R: AGGTTGTGGTCTGCACACTG	BC075014.2
*p65NFkB*	F: CAGAAGCAGGCTGGAGGTAA	R: GTTAGGCACAGGGACAATGC	L19067.1
*KEAP1*	F: TTCAGCTACACCCTGGAGGA	R: CTTGAAGACAGGGCTGGATG	BC002417.2
*NRF2*	F: ACACGGTCCACAGCTCATC	R: TGCCTCCAAAGTATGTCAATCA	BC011558.1
*NOX4*	F: CTCAGCGGAATCAATCAGCTGTG	R: AGAGGAACACGACAATCAGCCTTAG	BC040105.1

## Data Availability

Data is contained within the article or supplementary material.

## Supplementary Materials

Supplementary Materials Supplementary Figure of iNOS and Actin Western Blot (Figure 3)
