# Supplementary figures and images for: Short-Term In Vitro ROS Detection and Oxidative Stress Regulators in Epiretinal Membranes and Vitreous from Idiopathic Vitreoretinal Diseases
Source: Biomed Res Int. 2022 Dec 16;2022:7497816. doi: 10.1155/2022/7497816 (PMC9788888; doi:10.1155/2022/7497816)

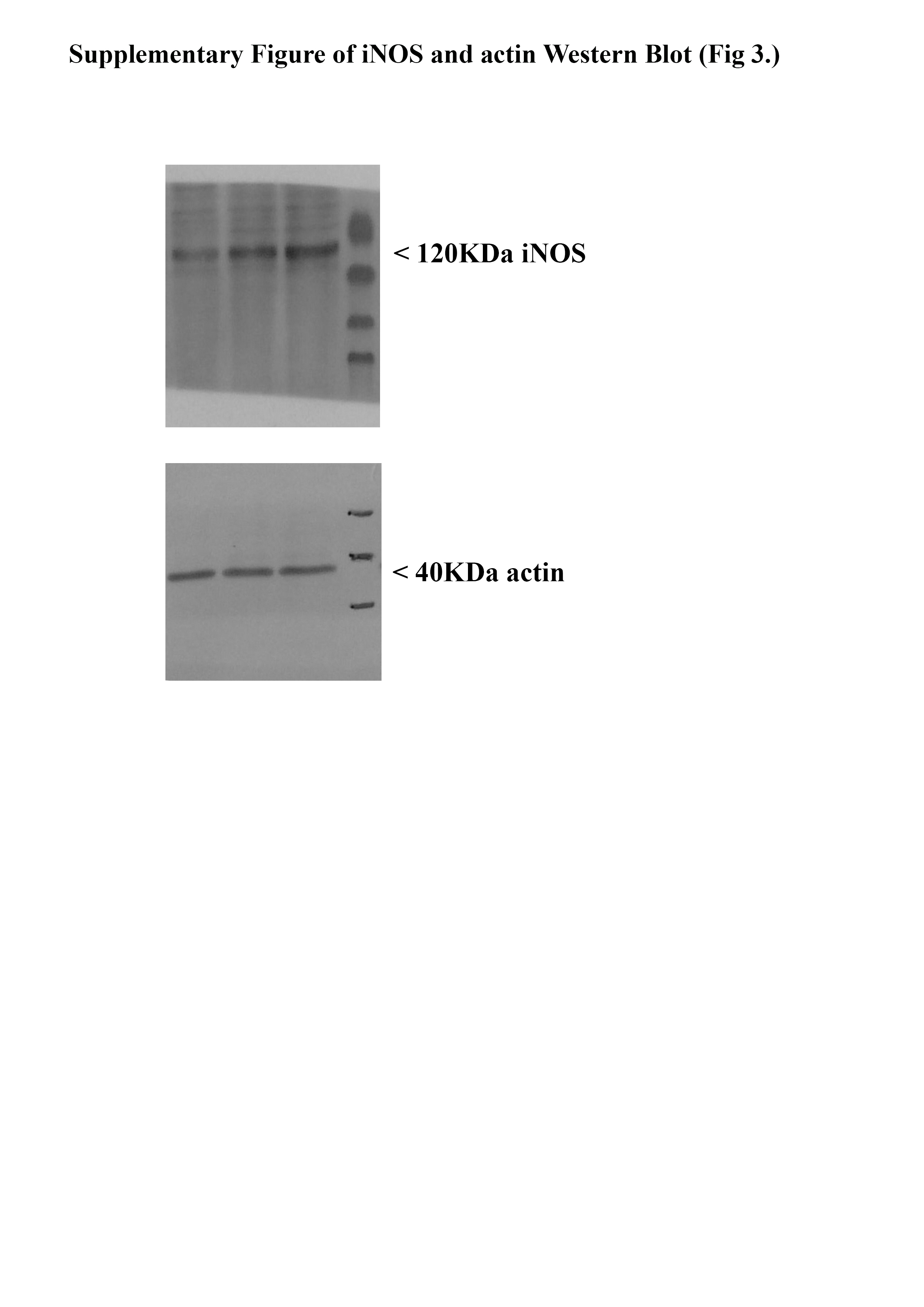

Supplement: Supplementary Materials — Supplementary Figure of iNOS and Actin Western Blot (Figure 3) [file 7497816.f1.jpg]
